# The Role of miR-181c in Mechanisms of Diabetes-Impaired Angiogenesis: An Emerging Therapeutic Target for Diabetic Vascular Complications

**DOI:** 10.3389/fphar.2021.718679

**Published:** 2021-08-13

**Authors:** Emma L. Solly, Peter J. Psaltis, Christina A. Bursill, Joanne T. M. Tan

**Affiliations:** ^1^ Vascular Research Centre, Lifelong Health Theme, South Australian Health and Medical Research Institute, Adelaide, SA, Australia; ^2^ Adelaide Medical School, The University of Adelaide, Adelaide, SA, Australia; ^3^ ARC Centre of Excellence for Nanoscale BioPhotonics, The University of Adelaide, Adelaide, SA, Australia

**Keywords:** hypoxia, endothelial dysfunction, migration, proliferation, apoptosis, mitochondrial function, axon guidance

## Abstract

Diabetes mellitus is estimated to affect up to 700 million people by the year 2045, contributing to an immense health and economic burden. People living with diabetes have a higher risk of developing numerous debilitating vascular complications, leading to an increased need for medical care, a reduced quality of life and increased risk of early death. Current treatments are not satisfactory for many patients who suffer from impaired angiogenesis in response to ischaemia, increasing their risk of ischaemic cardiovascular conditions. These vascular pathologies are characterised by endothelial dysfunction and abnormal angiogenesis, amongst a host of impaired signaling pathways. Therapeutic stimulation of angiogenesis holds promise for the treatment of diabetic vascular complications that stem from impaired ischaemic responses. However, despite significant effort and research, there are no established therapies that directly stimulate angiogenesis to improve ischaemic complications such as ischaemic heart disease and peripheral artery disease, highlighting the immense unmet need. However, despite significant effort and research, there are no established therapies that directly stimulate angiogenesis in a clinical setting, highlighting the immense unmet need. MicroRNAs (miRNAs) are emerging as powerful targets for multifaceted diseases including diabetes and cardiovascular disease. This review highlights the potential role of microRNAs as therapeutic targets for rescuing diabetes-impaired angiogenesis, with a specific focus on miR-181c, which we have previously identified as an important angiogenic regulator. Here we summarise the pathways currently known to be regulated by miR-181c, which include the classical angiogenesis pathways that are dysregulated in diabetes, mitochondrial function and axonal guidance, and describe how these relate both directly and indirectly to angiogenesis. The pleiotropic actions of miR-181c across multiple key angiogenic signaling pathways and critical cellular processes highlight its therapeutic potential as a novel target for treating diabetic vascular complications.

## Introduction

Diabetes mellitus (DM) currently affects up to 463 million people worldwide, imposing significant health and economic (USD 760 billion p.a., 12% of global health expenditure) burden (International Diabetes Federation, 2019). These numbers will continue to rise and are expected to reach 700 million people by the year 2045, making it the fastest growing global health epidemic (Taylor et al., 2013). The leading cause of morbidity and mortality in people with diabetes is attributable to its vascular complications (Abaci et al., 1999). These stem from impaired vascular function caused by chronic hyperglycaemia. This leads to higher instances of macrovascular diseases such as atherosclerosis, which includes coronary artery disease (CAD), peripheral artery disease (PAD) and cerebrovascular disease. Microvascular pathologies, including retinopathy, nephropathy, and neuropathy are also elevated. Patients with atherosclerotic occlusions also experience poor neovascularisation responses, which leads to worse clinical outcomes. Cutaneous wound healing is also impaired (Abaci et al., 1999). Individuals with type 2 diabetes mellitus (T2DM) have a 2-fold increase in all-cause mortality and a 3-fold increase in the risk of myocardial infarction (MI) (Willyard, 2012). Furthermore, when diagnosed with PAD associated with critical limb ischaemia (CLI), individuals with T2DM have an 8-fold higher amputation rate which contributes significantly to patient morbidity (Ware and Simons, 1997). This leads to an increased burden on medical care, reduced quality of life and increased risk of early death. While therapies that target hyperglycaemia have improved management of the disease, patients with good glycaemic control still experience devastating vascular complications. This highlights the unmet clinical need for the discovery of new targets that effectively prevent the vascular complications of diabetes.

The micro- or macro-vascular complications of diabetes, while diverse, are often partly driven by endothelial cell dysfunction that leads to impaired angiogenic responses. Angiogenesis refers to the formation of new blood vessels from pre-existing ones. In different disease states, this process can be dysregulated leading to increased pathological inflammatory-mediated angiogenesis (Rezzola et al., 2020) and impaired physiological ischaemia-driven angiogenesis (Hazarika et al., 2007). Macrovascular complications are often mediated by a combination of both. For example, in established atherosclerotic plaques, inflammatory-driven plaque neovascularisation rapidly expands plaque size by acting as a conduit for inflammatory cells, growth factors and cytokines/chemokines. The expanding lesion restricts blood flow, creating ischaemia in the surrounding tissues. The neovascularisation response to tissue ischaemia is then impaired in diabetes. This regenerative ischaemia-driven angiogenesis response is critical for determining the long-term prognosis of a patient and will therefore form the focus of this review. Therapies that augment vessel growth have the potential to improve tissue perfusion and facilitate tissue repair and recovery, holding significant promise for the treatment of diabetic macrovascular complications. Despite showing positive pre-clinical outcomes, current strategies, which are often directed towards single gene targets, remain largely ineffective and have not achieved positive outcomes in a clinical setting (Ylä-Herttuala et al., 2017; Liew et al., 2020). This is likely owing to the complexities involved in these pathologies and because targeted regulation of a single pathway may be insufficient to overcome the pleiotropic effects of a diabetic milieu.

MicroRNAs (miRNAs) are small non-coding RNAs. They are implicated in almost all body systems and regulate the expression of multiple genes simultaneously through targeted inhibition of protein translation (O’Brien et al., 2018). miRNAs are emerging as powerful tools to treat complex diseases as they can target multiple genes, and regulate the expression of multiple proteins, therefore orchestrating control over diverse pathways at a functional level. Targeted regulation of miRNAs therefore presents as a promising therapeutic approach for diabetic vascular complications.

This review highlights the potential of microRNAs to be therapeutic targets that prevent diabetes-impaired ischaemia-driven angiogenesis. We have a specific focus on miR-181c, which we first identified as an important angiogenic regulator (Hourigan et al., 2018). Here we summarise the pathways currently known to be regulated by miR-181c which include the classical angiogenesis pathways that are dysregulated in diabetes, as well as cellular processes, such as axonal guidance and mitochondrial function. We describe how these relate both directly and indirectly to angiogenesis, providing the rationale for its therapeutic potential as a novel target for the rescue of diabetes-impaired angiogenesis.

## Angiogenesis and its Impairment in Diabetes

### Mechanisms of Ischaemia-Driven Angiogenesis

**FIGURE 1:**
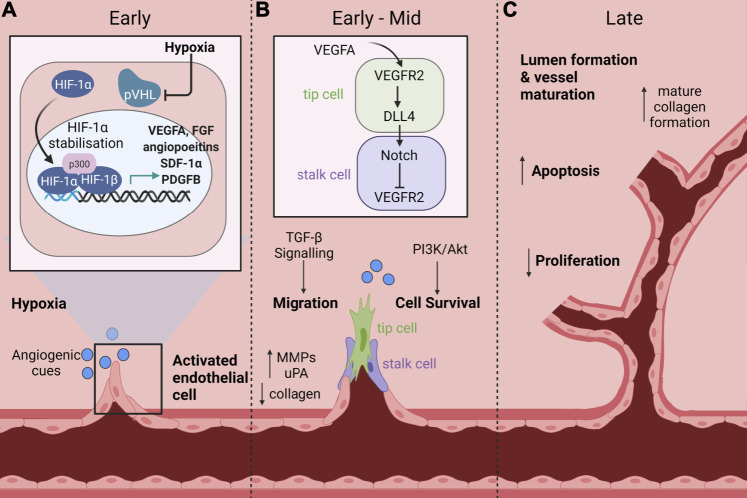
Signaling pathways and cellular processes that mediate ischaemia-driven angiogenesis. Ischaemia-driven angiogenesis involves several processes that work in a transient and overlapping fashion to allow new blood vessel formation. **(A)** Early: Endothelial cells (EC) become activated in response to an ischaemic injury. Under hypoxic conditions, the master regulator hypoxia inducible factor-1α (HIF-1α) is stabilised and protected from ubiquitin labelling by the von Hippel-Lindau (VHL) complex, preventing subsequent degradation. This allows HIF-1α to accumulate and translocate to the nucleus where it associates with HIF-1β. The HIF-1α/HIF-1β complex then binds to hypoxia response elements to induce the transcription of pro-angiogenic mediators including vascular endothelial growth factor A (VEGFA), fibroblast growth factor (FGF), angiopoietins, stromal cell derived factor-1α (SDF-1α) and platelet derived growth factor B (PDGFB). **(B)** Early-Mid: EC activation reduces the expression of collagen and increases the expression of proteolytic enzymes such as matrix metalloproteinases (MMPS), which promote degradation of the basement membrane and dissolution of adherent junctions that allow invasion of cells into the surrounding interstitial matrix. ECs are then specified into tip and stalk cells, which is controlled by Notch signaling. VEGFA stimulates tip cell induction and filipodia formation via VEGFR2, which promotes increased expression of the Notch ligand, delta-like ligand 4 (DLL4) in tip cells. Conversely, in adjacent cells, DLL4 promotes downregulation of VEGFR2, mediating a highly specified negative feedback loop between VEGFA and Notch to regulate tip and stalk cell formation. Additional signaling pathways including transforming growth factor-β (TGF-β) and phosphoinositide 3-kinase (PI3K)/Akt are activated to promote endothelial cell migration and survival respectively. **(C)** Late: As the lumen of the new vessel begins to form and the vessel matures, there is an increase in apoptosis and decrease in proliferation concomitant with an increase in mature collagen deposition. Created with BioRender.com.

Angiogenesis is an important process for normal growth and development during embryogenesis. It is also important for many physiological processes such as wound healing and for collateral vessel formation in response to vessel occlusion to help facilitate adaptive responses to low oxygen supply and injury. Angiogenesis can occur via two main mechanisms: sprouting and elongation, or intussusception. Intussusception is where vessel remodeling results in the splitting of the existing vessel. Both angiogenic mechanisms occur concurrently and are critical for tissue neovascularisation in response to ischaemia (Ware and Simons, 1997) and wound healing (Tonnesen et al., 2000). Angiogenesis requires several processes that work in a transient and overlapping fashion to allow new blood vessel formation and involves: basement membrane degradation, followed by endothelial cell (EC) proliferation, invasion and migration, tip and stalk cell specification, sprouting, elongation and anastomosis. The final stages involve other cell types such as smooth muscle cells and pericytes that assist with tissue remodeling and maturation (Figure 1) (Potente et al., 2011).

The master regulator in these processes is hypoxia inducible factor-1α (HIF-1α) which is stabilised in response to tissue ischaemia following, for example, MI or in CLI to increase angiogenesis (Fong, 2008). In normal oxygen environments, HIF-1α is continuously expressed in the cytoplasm and in the presence of oxygen is rapidly degraded (Fong, 2008). Rapid degradation of HIF-1α is mediated by prolyl hydroxylase (PHD) proteins. In the presence of oxygen, PHDs promote hydroxylation of proline residues on HIF-1α, which allows its recognition by the von Hippel-Lindau ubiquitin ligase complex (VHL) and subsequently targets HIF-1α for degradation (Semenza, 2010). In hypoxia, Siah1 and Siah2 are activated, which mediate PHD protein degradation, allowing HIF-1α stabilisation and accumulation in the cytoplasm. This in turn allows translocation of HIF-1α to the nucleus where it associates with HIF-1β, enabling it to bind to response elements on DNA. DNA binding induces transcription of angiogenic and metabolic expression programs, most notably the expression of vascular endothelial growth factor A (VEGFA), stromal cell derived factor-1α (SDF-1α), fibroblast growth factor (FGF), platelet derived growth factor B (PDGFB) and angiopoietins (Figure 1A) (Fong, 2008; Semenza, 2010).

Sprouting angiogenesis is the most widely investigated form of angiogenesis and involves the protrusion and elongation of specified stalk and tip cells from the endothelium towards angiogenic signaling cues (Figure 1). In the initial stages of sprouting angiogenesis ECs become activated by angiogenic mediators, such as VEGFA (Risau, 1997; Potente et al., 2011). EC activation increases the expression of proteolytic enzymes, such as matrix metalloproteinases (MMPS), which promote degradation of the basement membrane and dissolution of adherent junctions that allow invasion of cells into the surrounding interstitial matrix (Ferrara and Kerbel, 2005; Otrock et al., 2007). Following release, ECs, pericytes and vascular smooth muscle cells (VSMCs) proliferate and migrate towards chemoattractant signals. VEGFA induces cellular proliferation and migration through several pathways including mitogen-activated protein kinase (MAPK), extracellular signal-regulated kinase (ERK), p38 and c-Jun N-terminal kinase (JNK).

ECs are then specified into tip and stalk cells, which is controlled by Notch signaling (Figure 1B). VEGFA stimulates tip cell induction and filipodia formation via VEGF receptor 2 (VEGFR2), which promotes increased expression of the Notch ligand, delta-like ligand 4 (DLL4) in tip cells (Patan, 2000; Potente et al., 2011). Conversely, in adjacent cells, DLL4 promotes downregulation of VEGFR2, mediating a highly specified negative feedback loop between VEGFA and Notch to regulate tip and stalk cell formation. Tip cells function as a guide for vascular outgrowth in response to environmental cues which are received by guidance receptors; their function is analogous to axon growth (Potente et al., 2011). Stalk cells are more proliferative, forming tubes and branches which elongate and mature to eventually form the lumen. Following sufficient neovascularisation, the tissue enters a remodeling phase in which anti-angiogenic genes are expressed to reduce EC activation and promote vessel maturation. Proliferation is reduced and there is an increase in the expression of apoptosis related genes that allow lumen formation and vessel maturation. The vessel is then stabilised, which is aided through mature collagen formation and re-establishment of tight junctions and the basement membrane (Figure 1C) (Korn and Augustin, 2015). The process of ischaemia-mediated angiogenesis is critically regulated and is important for physiological processes such as collateral vessel formation in response to vessel occlusion and to facilitate healthy wound healing, processes which are often impaired by diabetes.

### Physiological Angiogenesis is Important for Collateral Vessel Formation

Vessel occlusions cause chronic imbalances in oxygen supply and demand. The resulting low levels of cellular oxygen induce adaptive neovascularisation in the ischaemic tissue. This facilitates the growth of collateral vessels that redirect blood supply around the arterial occlusions that occur in CLI and MI, which prevents tissue necrosis in the affected sites of the lower leg and heart (Collinson and Donnelly, 2004).

Diabetic patients experience higher rates of PAD and MI and are more likely to die as a result (Taylor et al., 2013). In addition to this, recovery post ischaemia is impaired in diabetes, stemming from an inability to form new collateral vessels, leading to tissue death and resulting in poor patient recovery and survival (Abaci et al., 1999). Patients with T2DM experience reduced coronary collateral formation following vascular occlusion, due to impaired angiogenesis responses to tissue ischaemia. In cardiomyocytes following MI, impaired collateral formation can lead to exacerbated ischaemic injury. This promotes cell death and resulting in higher rates of patient mortality following MI (Kalogeris et al., 2012). In CLI, impaired induction of angiogenesis contributes to higher rates of lower limb amputation in patients with diabetes. Surgical intervention is a common treatment for CLI (Shammas, 2007) in which the occlusions are removed via a number of different procedures. Unfortunately, some patients with T2DM are not suitable candidates and have no option other than amputation. This has a significant impact on quality of life (Ouma et al., 2012).

### Physiological Angiogenesis is Critical for Wound Healing

In addition to poor collateral vessel formation in response to vessel occlusion, impaired wound healing in diabetes also contributes significantly to higher rates of lower limb amputations in these patients. While this process is complex, it is also characterised by impaired ischaemia-driven angiogenesis, which is discussed in more detail below.

The wound healing process comprises the cooperation of distinct yet overlapping phases of inflammation, proliferation, contraction, and tissue remodeling (Braiman-Wiksman et al., 2007). Growth factors and other signaling molecules are released that mediate the response to injury and promote the culmination of cellular processes of migration, proliferation, and differentiation to facilitate re-epithelialisation, re-vascularisation, contraction, nerve innervation, and scar formation leading to wound repair. Platelet aggregation and granulation precedes clot formation in response to injury to ensure rapid haemostasis (Braiman-Wiksman et al., 2007). Platelets release several growth factors and inflammatory cytokines which begin the wound healing processes including PDGF, TGF-β, EGF and FGF (Werner and Grose, 2003).

Hypoxia is essential for normal wound healing, where it drives the induction of HIF-1α to facilitate transcription of critical mediators of tissue repair, including angiogenesis (Botusan et al., 2008). Angiogenesis occurs during all stages of wound repair to create new microvascular networks (Honnegowda et al., 2015). In the early stages of wound healing angiogenesis aids in the recruitment of endothelial progenitor cells, macrophages, keratinocytes and fibroblasts which facilitate re-epithelialisation and wound repair (Gurtner et al., 2008; Johnson and Wilgus, 2014). Keratinocytes migrate across the wound edge, independent of granulation tissue formation, and this forms one of the initial steps in the wound healing process (Braiman-Wiksman et al., 2007). Inflammatory cells like macrophages are recruited within the granulation tissue and release additional cytokines and angiogenic factors like fibroblast growth factor (FGF) and tumour necrosis factor-α (TNFα) (Gurtner et al., 2008). Following the initial inflammatory phase, the tissue enters a proliferative phase to help facilitate wound closure. Proliferation of the epidermal leading edge composed of both proliferating and migrating cells like fibroblasts occurs coordinately with differentiation of the epidermis (Gurtner et al., 2008; Honnegowda et al., 2015). After the initial phase of proliferation, granulation tissue formation progresses followed by the final phase in wound healing: tissue remodeling (Reinke and Sorg, 2012).

Diabetic foot ulcers (DFU) are one of the major causes of lower limb amputations in patients with diabetes and are perpetuated by chronic non-healing wounds. These chronic non-healing wounds precede 85% of limb amputations and cause significant reductions in the quality of life for patients (Elgzyri et al., 2013). These issues are further exacerbated in diabetes due to an impairment in the angiogenic response to wound ischaemia that significantly delays healing (Järbrink et al., 2017). In diabetic mice, delayed wound healing is associated with reduced angiogenesis, which is characterised by reduced capillary density and delayed granulation tissue formation (Michaels et al., 2007; Botusan et al., 2008). This is preceded by reduced stabilisation and activation of HIF-1α within the wound environment (Botusan et al., 2008), leading to impaired induction of angiogenesis signaling molecules, like VEGFA.

Whilst pre-clinical studies have found that angiogenic stimulation with VEGFA improves wound healing in diabetic mice (Galiano et al., 2004), this failed to translate in a Phase II clinical trial assessing the healing of diabetic foot ulcers (Wietecha and DiPietro, 2013). This lack of clinical efficacy suggests that the mechanisms of diabetes-related vascular complications are complex and not fully understood, emphasising a need for further research.

The next section will highlight what is currently known about the mechanisms surrounding diabetes-impaired angiogenesis in responses that contribute to impaired collateral vessel formation and poor wound healing.

### Mechanisms of Diabetes-Impaired Angiogenesis in Response to Ischaemia

Physiological ischaemia-driven angiogenesis requires a delicate balance between pro-angiogenic and anti-angiogenic factors. Dysregulation of this balance leads to diseased states, such as that seen in patients with diabetes.

Impaired angiogenic responses to tissue ischaemia are central to the reduced capacity of diabetic patients to recover following vascular events and injury (Abaci et al., 1999; Okonkwo and DiPietro, 2017). While the mechanisms that underpin diabetes-impaired angiogenesis have not been fully elucidated, current understanding suggests that this stems from hyperglycaemia-induced production of advanced glycation end-products (AGEs), which contribute to endothelial dysfunction (Soro-Paavonen et al., 2010). This leads to the dysregulation of the classical angiogenesis pathways and signaling molecules. HIF-1α stability is reduced in diabetic mice (Mace et al., 2007; Botusan et al., 2008) and reduced circulating HIF-1α protein expression is seen in diabetic patients (Pichu et al., 2018). Higher incidences of *HIF1A* gene polymorphisms in diabetic patients are associated with higher rates of diabetic foot ulcers (Pichu et al., 2018). Subsequently, decreased VEGFA production and signaling via VEGFR2 play a central role to reduce blood flow reperfusion in diabetes (Rivard et al., 1999). This is characterised by reduced neovascularisation to the ischaemic limbs of diabetic mice (Rivard et al., 1999). Additionally, endothelial dysfunction in diabetes leads to impaired metabolic activity (Sas et al., 2016) and inhibition of endothelial nitric oxide synthase (eNOS) activity (Lin et al., 2002). Hyperglycaemia triggers accumulation of reactive oxygen species (ROS) and imbalances to nitric oxide (NO) bioavailability, leading to reduced eNOS, and abating the protective effects of NO on vascular tone, thrombosis, immune responses, and vascular repair mechanisms (Tahergorabi and Khazaei, 2012). In a hyperglycaemic environment, AGE accumulation promotes activation of the AGE receptor (RAGE), which activates ROS-sensitive biochemical pathways and in turn dysregulates metabolic signaling in addition to promoting oxidative stress and inflammation via the transcription factor nuclear factor-κB (NF-κB) (Tahergorabi and Khazaei, 2012). These impaired systems play a role in the development of vascular complications in diabetes.

Therapeutic stimulation of angiogenesis holds promise for the treatment of ischaemic diseases, but current therapies lack clear clinical efficacy (Ylä-Herttuala et al., 2007). This highlights the need for the identification of novel treatment targets and the development of new therapies to overcome these devastating effects.

## MicroRNAs as Therapeutic Targets for Diabetic Vascular Complications

microRNAs are small non-coding RNAs, 18–22 nucleotides in length. They were first discovered in 1993 by the Ambros and Ruvkun laboratories in *C.Elegans* and since then have transformed our understanding of gene expression, regulation and function (Lee et al., 1993; Wightman et al., 1993)*.* During canonical biogenesis, which is under strict regulatory control, miRNAs are transcribed by RNA polymerase II into a double-stranded hairpin primary transcript called pri-miRNA. Pri-miRNAs are cleaved by the enzyme Drosha generating precursor miRNA termed pre-miRNA (O’Brien et al., 2018). Pre-miRNA is exported to the cytoplasm and then undergoes further processing by the RNase III endonuclease Dicer (O’Brien et al., 2018). Dicer removes the terminal loop of pre-miRNA producing mature miRNA duplex strands, termed -5p or -3p. Either one of these strands can bind with the Argonaute proteins to form what is referred to as the RNA-induced silencing complex (RISC) (Khvorova et al., 2003). Once forming RISC, miRNAs regulate protein translation through complementary base pairing to the 3’untranslated region (UTR) of their mRNA targets (Ha and Kim, 2014). This integration of mRNA into the miRNA-RISC complex facilitates enzymatic cleavage or deadenylation of the target mRNA promoting its degradation or blocking protein translation (Vasudevan, 2012).

miRNAs have been shown to play a role in regulating numerous cellular processes, including angiogenesis and their dysregulation has been shown to contribute to disease progression (Yilmaz et al., 2018). miRNAs can control the regulation of numerous genes simultaneously and have very context and cell specific roles. These context-specific roles are thought to be dependent on varying layers of miRNA regulation (Liu et al., 2008; Jee and Lai, 2014). Given the vast impact of miRNAs on gene expression, robust control over their regulation is essential, and this control is often impaired in disease states. miRNAs can undergo various pre- and post-transcriptional modifications, but their activity can also be manipulated by other non-coding RNAs, such as long noncoding RNAs (lncRNAs) and circular RNAs (cirRNAs) (Zhao, 2018).

The global importance of miRNAs has been well established and their dysregulation is prominent in numerous diseases. Global knockout of key miRNA machinery, like Dicer, results in embryonic lethality in mice (Bernstein et al., 2003) and is also required for angiogenesis during development (Yang et al., 2005). Dysregulation of Dicer expression is also prevalent in disease (Zhang et al., 2014). In diabetes, hyperglycaemia reduces Dicer expression. Additionally, lack of Dicer1 in beta-cells leads to impaired insulin signaling and promotes the development of diabetes (Kalis et al., 2011). During wound repair Dicer is transiently regulated to facilitate wound healing. However, in diabetic mice the timing of Dicer regulation is altered, contributing to impaired wound repair (Mahdipour and Hasanzadeh, 2017). These changes in Dicer expression have significant effects on miRNA function and expression. In addition to Dicer, many other molecules involved in miRNA control are dysregulated in disease, including Argonaute proteins and assembling of the RISC complex (Lewkowicz et al., 2015). One of the major determinants of miRNA function are Argonaute proteins and these are critical for normal cellular function and survival. These proteins undergo various modifications that translate to alterations to miRNA activity including phosphorylation, ubiquitination and PARylation (Jee and Lai, 2014). Argonaute proteins can also undergo modification induced by hypoxia. Upregulation of hypoxia-inducible prolyl-4-hydroxylases that promote human Argonaute-2 hydroxylation promotes increases in miRNA levels and activity (Wu et al., 2011).

Changes to miRNA expression profiles is highly implicated in disease and leads to altered functionality of important biological processes (Solly et al., 2019). In addition, miRNAs have been shown to be novel targets for mediating pro- or anti-angiogenic effects (Landskroner-Eiger et al., 2013). For example, overexpression of the pro-angiogenic miR-27b improves ischaemia-mediated neovascularisation (Veliceasa et al., 2015) and wound healing (Wang et al., 2014). Conversely, inhibition of the anti-angiogenic miR-92a enhanced neovascularisation in mouse models of hindlimb ischaemia and MI (Bonauer et al., 2009). Targeted modulation of miRNAs is clinically feasible and may be more effective because the orchestration of a host of complex factors may be required to therapeutically stimulate angiogenesis in a diabetic setting. Currently, miRNAs are in Phase I and II clinical trials for the treatment of scleroderma, cancer and hepatitis C virus (Mellis and Caporali, 2018) in which they are showing promising results. This highlights the great potential of miRNAs as therapeutic targets for complex multifaceted diseases. Due to the pleiotropic action of miRNAs their modulation may offer more promising therapeutic benefits for diabetic vascular complications.

## MicroRNA-181 Family

The miR-181 family consists of four members that are highly conserved across vertebrates: miR-181a, -181b, -181c and -181d (Dostie et al., 2003; Lagos-Quintana et al., 2003; Lim et al., 2003; miRBase, 2018). MiR-181a/b cluster together at two different genomic locations on chromosome 1 (miR-181a1/b1) and chromosome 9 (miR-181a2/b2) and miR-181c/d cluster together on chromosome 19 (Dostie et al., 2003; Lagos-Quintana et al., 2003; Lim et al., 2003). miRNA transcripts are often transcribed in tandem with key genes involved in their regulation or function. However, within the chromosomal regions of the miR-181 transcripts no other protein coding regions are found, indicating that these miRNAs are transcribed independently (Dostie et al., 2003; Lagos-Quintana et al., 2003; Lim et al., 2003). The -3p and -5p strands of each miR-181 family member differ from each other and have modestly varying seed sequences (Dostie et al., 2003; Lagos-Quintana et al., 2003; Lim et al., 2003). The sequences of the -5p strands differ by only a few nucleotides across the different family members. However, these share identical seed regions. Despite this similarity, each member exhibits distinct gene targets, which confers varied functionality and context-specific functions. miR-181 family members have been highly implicated in inflammation and several cancers, often having both oncogenic and tumour suppressive properties depending on specific cancer types (Sun et al., 2014; Pop-Bica et al., 2018). miR-181c expression has been shown to be dysregulated in several cancers including inflammatory breast cancer (Zhang and Zhang, 2015), gastric cancer (Hashimoto et al., 2010), pancreatic cancer (Chen et al., 2015), brain cancer (Tominaga et al., 2015), lung cancer (Zhang et al., 2017) and colon cancer (Yamazaki et al., 2017). Dysregulation of miR-181c has vast effects on tumourigenesis, affecting the proliferative, survival and migratory capacity of cancer cells (Li et al., 2014; Ruan et al., 2015).

**FIGURE 2:**
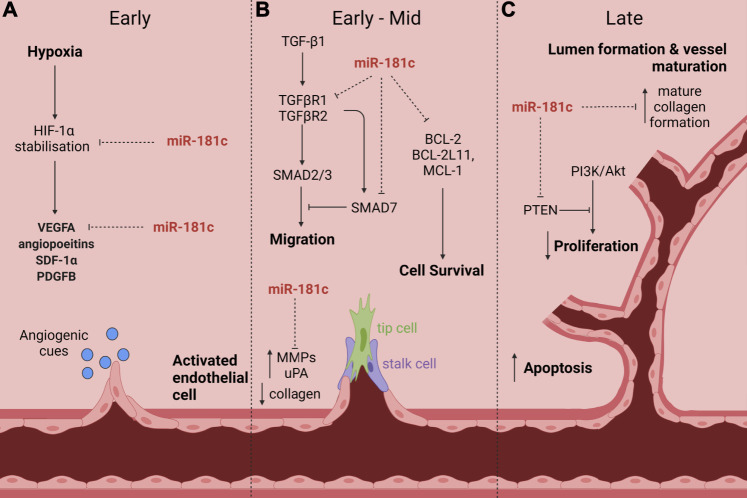
The proposed role of miR-181c action in diabetes-impaired angiogenic responses to ischaemia. Numerous studies have shown that miR-181c targets many signaling pathways and cellular processes that are critical in diabetes-impaired angiogenic responses to ischaemia. This includes **(A)** HIF-1α stabilisation and VEGFA expression in the early phase; **(B)** TGF-β signaling (via TGFβR1, TGFβR2 and SMAD7) to inhibit cell migration, BCL-2 and its family members BCL-2L11 and MCL-1 which drive cell survival and MMPs and uPA in the early-mid phase; and **(C)** PTEN and dysregulation of collagen in the late phase. Created with BioRender.com.

We have identified, for the first time, that miR-181c has anti-angiogenic properties (Hourigan et al., 2018). We will discuss some of the pathways by which miR-181c regulates angiogenesis and highlight why it is a promising targeting for treating diabetes impaired-angiogenesis (Figure 2).

## The Role of MicroRNA-181c in Diabetes-Impaired Angiogenesis

As previously discussed, impaired angiogenesis underpins the development of vascular complications and impaired wound healing in diabetes. This arises due to hyperglycaemia-induced endothelial dysfunction and the impairment of signaling pathways and molecules in response to high glucose. Dysregulation of miR-181c by hyperglycaemia may contribute to these effects. Expression of miR-181c has been shown to be dysregulated in human umbilical vein endothelial cells (HUVECs) in response to high glucose exposure (Zitman-Gal et al., 2014; Shen et al., 2018), which is meant to mimic a diabetic-like environment *in vitro*. Overexpression of miR-181c significantly increased ROS production in HUVECs treated with high glucose and enhanced high glucose-induced oxidative stress and cell death (Shen et al., 2018). Increases in ROS production contribute to endothelial activation and precede vascular dysfunction in diabetes, suggesting that dysregulation of miR-181c in response to high glucose may contribute to hyperglycaemia-induced endothelial cell damage. This may also play a contributing role in diabetes-impaired angiogenesis responses, which will now be discussed in more detail.

### Tubule Formation and the Expression of Critical Angiogenesis Mediators

The role of miR-181c in angiogenesis is complex and currently not well understood. We were the first to show that miR-181c has anti-angiogenic properties (Hourigan et al., 2018). We found that overexpression of miR-181c in endothelial cells inhibited endothelial vascular network formation and inhibited protein levels of the pro-angiogenic mediator VEGFA (Hourigan et al., 2018). Conversely, miR-181c inhibition promoted tubule formation and VEGFA expression (Hourigan et al., 2018). While there has not been a direct link between miR-181c and diabetes-impaired angiogenesis to date, we have previously shown that inhibition of miR-181c may mediate the pro-angiogenic effects of high-density lipoproteins (HDL) in diabetes (Hourigan et al., 2018). We have shown that HDL rescues diabetes-impaired angiogenesis via the classical HIF-1α/VEGFA signaling axis (Castelli et al., 1986; Vickers et al., 2011; Tan et al., 2014). Furthermore, we have demonstrated that the pro-angiogenic effects of HDL in diabetes may be mediated, in part, through its interaction with miR-181c (Hourigan et al., 2018). Infusions of HDL into diabetic mice significantly reduced miR-181c levels in the tissue early post-ischaemia when angiogenic responses are important. This was associated with improved blood flow reperfusion to the ischaemic hindlimb (Hourigan et al., 2018). This suggests that miR-181c inhibition may mediate the pro-angiogenic action of HDL and provide an alternate strategy to rescue diabetes-impaired angiogenic responses to ischaemia.

HDL can also carry endogenous miRNAs, transporting them to specific tissues in the body to impart functional effects (Castelli et al., 1986; Vickers et al., 2011; Tan et al., 2014). Circulating miRNAs have been found to correlate with diabetes-associated microvascular complications (Wang et al., 2016). Low HDL levels are an independent risk factor for the development of T2DM (von Eckardstein et al., 2000) and are associated with an increased risk of diabetes-associated microvascular complications (Morton et al., 2018). We recently found that HDL-bound miR-181c levels are strikingly elevated in Indigenous Australian males with diabetes-associated peripheral artery disease, a population that is disproportionately impacted by diabetic vascular complications (Morrison et al., 2021). HDL becomes dysfunctional in a diabetic environment, losing its vasculo-protective properties and becoming pro-atherogenic (Femlak et al., 2017). Treatment of HCAECs with extracted HDL from these individuals resulted in impaired tubule formation and an inability to induce HIF-1α expression (Morrison et al., 2021). This suggests that higher naturally occurring amounts of HDL-bound miR-181c may result in dysfunctional HDL and contribute to the loss of its pro-angiogenic effects in diabetes.

miR-181c has also been shown to be divergently regulated by hypoxia in culture (Hourigan et al., 2018; Deng et al., 2020). In human coronary artery endothelial cells (HCAECs), hypoxia reduced miR-181c expression (Hourigan et al., 2018), whereas in eye vascular endothelial cells (VECs) miR-181c levels were increased in response to hypoxia exposure (Deng et al., 2020). We showed that miR-181c inhibition increased tubule formation of HCAECs by promoting increased VEGFA protein expression; a well-established pro-angiogenic growth factor and HIF-1α inducible gene (Hourigan et al., 2018). In addition, stabilisation of the master regulator HIF-1α can be mediated by miR-181c. In breast cancer cells exposed to hypoxia, overexpression of miR-181c suppressed HIF-1α stabilisation and this led to a reduction in hypoxia-inducible glycolysis related enzymes (Lee et al., 2019). Conversely, in eye vascular ECs (VECs) which are prone to inflammatory-driven angiogenesis in diabetes, miR-181c was shown to promote stabilisation of HIF-1α in these cells (Deng et al., 2020).

These studies demonstrate that miR-181c has divergent roles in response to hypoxia in diabetes, which are largely dependent on the cell type. In cells more prone to increased inflammatory-driven angiogenesis in diabetes, miR-181c promotes angiogenesis. Whereas, in cells that experience decreased ischaemia-driven angiogenesis in diabetes, the opposite effect is seen, and miR-181c reduces angiogenesis in this context. The divergent regulation of miR-181c in response to hypoxia and high glucose in different cell types and the varying effects of miR-18c on HIF-1α stabilisation and subsequently tubule formation highlights the context specific role of this miRNA under varying conditions. [deleted text] While the role of miR-181c on ischaemia-driven angiogenesis in diabetes is yet to be fully determined, these studies suggest that miR-181c targets ischaemia-mediated genes that play a crucial role in this process. Functionally, *in vitro*, *in vivo* and *ex vivo* experiments suggest an important role for miR-181c in tubule formation and that targeting this miRNA rescues diabetes-impaired responses in different contexts.

In addition to these critical regulators and the formation of endothelial tubules, there are multiple facets that contribute to the process of angiogenesis that may also be regulated by miR-181c. These include basement membrane degradation, cellular proliferation, invasion and migration, apoptosis, cell survival and mitochondrial function as well as tissue remodeling and maturation (Figure 2). These processes are orchestrated in a cohesive manner to facilitate effective blood vessel growth and function (Potente et al., 2011). The potential role of miR-181c in these important processes that help to facilitate ischaemia-driven angiogenesis is discussed in more detail below.

### Basement Membrane Degradation, Tissue Remodeling and Maturation

An important first step in angiogenesis is an increase in EC permeability via dissolution of tight junctions and degradation of the basement membrane to allow invasion and escape of cells into the interstitial matrix. Integral to these processes are MMPs which facilitate breakdown of these components (Potente et al., 2011). In addition to promoting extracellular matrix (ECM) degradation, MMPs also facilitate the release of ECM-bound angiogenic signaling cues and contribute to the formation of angiogenic concentration gradients within the matrix (Löffek et al., 2011). MMPs facilitate detachment of pericytes allowing vascular plasticity and growth (Rundhaug, 2005), but also contribute to a vast extracellular signaling network (Löffek et al., 2011). In the later stages of angiogenesis and wound healing, synthesis of the ECM promotes tissue remodeling and maturation of vessels through coordinated regulation from pro- and anti-fibrosis related cytokines (Löffek et al., 2011). miR-181c has been reported to play an important role in a number of these processes.

Urokinase type plasminogen activator (uPA) regulates angiogenesis and vascular permeability through proteolytic degradation of the ECM but can also act as a transcription factor that regulates VEGFR2 transcription (Stepanova et al., 2016). miR-181c inhibition increased uPA expression as well as the matrix protein MMP1 in hypertrophic scars (Li et al., 2015). However, inhibition of miR-181c has no effect on MMP1 expression under normal conditions, highlighting that miR-181c can target matrix genes that are involved in angiogenesis (Stepanova et al., 2016). This indicates that coordinated regulation of miR-181c activity may be important for containing an appropriate balance of matrix protein expression during angiogenesis. Inhibition of miR-181c also resulted in decreased collagen 1 (*COL1*) expression (Li et al., 2015). Excessive collagen deposition can lead to hypertrophic scar formation and impair the wound healing process (Xue and Jackson, 2015). In the early stages of wound healing collagen formation is premature and non-structured. However, in the later stages collagen matures into well organised fibers, facilitating normal wound closure (Xue and Jackson, 2015). As inhibition of miR-181c reduced collagen expression in hypertrophic scars, this suggest that miR-181c may also play a role in dysregulating collagen maturation in wounds, which can severely impede wound closure.

In addition to targeting genes that promote ECM destruction in the skin, miR-181c is excreted from brain cancer cells within extracellular vesicles that promote blood-brain barrier destruction. These vesicles have been implicated in facilitating abnormal actin localisation (Tominaga et al., 2015). This occurs through miR-181c-induced degradation of 3-phosphoinositide-dependent protein kinase-1 (PDPK1), which dysregulates actin organisation. Breakdown of this blood-brain barrier is conducive to brain metastasis and highlights a role for miR-181c in the breakdown of endothelial cell layers, facilitating cellular migration, and in cancer, may promote metastasis.

These studies highlight a potential role for miR-181c in the initial phases of angiogenesis and wound healing (Figure 2). These demonstrate its potential for the breakdown of endothelial cell layers, which aid in the release of ECs, pericytes and VSMC into the interstitial space, facilitating budding and extension of sprouting vessels through the ECM. Additionally, miR-181c may also contribute to the final stages of angiogenesis and wound healing by mediating mature collagen deposition and tissue remodeling.

### Cellular Proliferation, Apoptosis and Survival

Both proliferation and apoptosis are essential processes that contribute to angiogenesis. Early cellular proliferation is important in facilitating budding and extension of cells from existing vessels into the surrounding interstitial space (Potente et al., 2011). Apoptosis, or programmed cell death, is inherently a survival response but is also regulated by physiological processes to ensure normal cell growth. There are two main pathways of apoptosis, the extrinsic or death receptor pathway, and the intrinsic or mitochondrial pathway (Elmore, 2007). Apoptosis and proliferation are transiently regulated to facilitate processes such as angiogenesis. In the early stages of angiogenesis apoptosis is reduced to support cell growth and proliferation of budding cells, but upon vessel maturation there is an increase in apoptosis related genes which aid in inhibiting angiogenesis when it is no longer required. Several angiogenesis signaling molecules can inhibit apoptosis and promote cell survival including VEGFA, angiopoietin 1, and FGF, which occurs primarily through activation of the PI3K/Akt pathway (Alon et al., 1995; Gerber et al., 1998; Fujio and Walsh, 1999; Hayes et al., 1999; Kwak et al., 1999; Papapetropoulos et al., 2000). Dysregulation of apoptosis and proliferation are hallmarks of many cancers, which contributes to cell immortalisation but also aids in facilitating tumour angiogenesis. Additionally, apoptosis becomes dysregulated in diseases like diabetes, where it is associated with high glucose-induced cell death of beta-cells. In HCAECs, high glucose-induced apoptosis involves the upregulation of death receptors like tumor necrosis factor receptor 1 (TNFR1) and Fas (Kageyama et al., 2011). In the vasculature dysregulated apoptosis plays a role in atherosclerosis and induces cell death in response to ischaemia reperfusion injury following myocardial infarction.

In the rat myocardium, miR-181c levels are increased in response to ischaemia-reperfusion injury (Ge et al., 2019). Overexpression of miR-181c in cultured cardiomyocytes promotes increased expression of apoptosis related genes including caspsase-3 and bax/Bcl-2, leading to an increase in apoptosis (Ge et al., 2019). In inflammatory breast cancer, miR-181c is overexpressed and acts as an oncogene, promoting proliferation and cell survival by inhibiting the tumour suppressor gene PTEN (Zhang and Zhang, 2015). PTEN negatively regulates the PI3K/Akt pathway to inhibit proliferation. miR-181c has also been shown to target PTEN in endometrial cancer (Zhuang et al., 2019), potentiating cell survival through reduced apoptosis. In response to hypoxia, suppression of miR-181c promoted cardiomyocyte survival by increasing phosphorylation of Akt and also upregulating the cell survival mediator, Bcl-2 (Li X. et al., 2020). An increase in Bcl-2 protein expression was also seen in HUVECs following miR-181c inhibition and resulted in reduced high glucose-induced apoptosis (Shen et al., 2018). In addition to Bcl-2, miR-181c was also shown to directly target other Bcl-2 family members in astrocytes, including Bcl-2L11 and Mcl-1 (Ouyang et al., 2012).

These studies demonstrate a regulatory role for miR-181c in both apoptotic and cell survival related genes (Figure 2). miR-181c can mediate the coordinated expression of pro- and anti-apoptotic genes, and additionally regulates one of the main proliferative pathways perturbed in cancer and diabetes: PI3K/Akt. The ability of miR-181c to mediate cellular survival and death may contribute to its pleiotropic effects on impaired angiogenesis in diabetes.

### Cellular Invasion and Migration

Cellular migration is integral to adequate angiogenesis as it supports sprouting of tip and stalk cells through the ECM, which forms the new vessel (Lamalice et al., 2007). In several cancers alterations in miR-181c levels have been shown to regulate cellular migration through regulation of Notch and TGF-β1 signaling (Li et al., 2014; Ruan et al., 2015; Fu et al., 2019).

The TGF-β pathway has been extensively implicated in regulating numerous cellular functions including actin polymerisation, cell adhesion, angiogenesis, ECM neogenesis, immunosuppression, apoptosis induction, as well as cell cycle and differentiation (Derynck and Zhang, 2003; Vander Ark et al., 2018). Active TGF-β ligands bind to TGF-β receptor type I (TGFβR1) and II (TGFβR2) promoting receptor phosphorylation. This promotes activation of the SMAD pathway. SMAD proteins act as transcription factors that mediate gene expression. R-Smads are phosphorylated, which then bind to common Smad mediators (SMAD4) to form R-Smad/Co-Smad complexes. These are then able to translocate into the nucleus and promote gene expression through DNA binding. R-Smad/Co-Smad DNA binding induces transcription of genes involved in several important cellular processes (Vander Ark et al., 2018). Crosstalk between TGF-β and a myriad of signaling pathways involved in angiogenesis, wound healing and axon development implicates TGF-β prominently as a mediator of diabetes-impaired responses. There is strong evidence to suggest that TGF-β is one of the major molecules contributing to diabetic kidney disease (Zhao et al., 2020). TGF-β dysregulation in diabetes may also alter wound healing. Improved remodeling of wounds was found to be associated with reduced TGF-β expression in the late stages of healing (Fekrazad et al., 2018). miR-181c has been implicated as a regulator of the TGF- β pathway.

Reduced expression of miR-181c was identified in glioblastoma (He et al., 2016) and osteosarcoma (Fu et al., 2019) and is associated with poor clinical prognosis of these cancers (Mori et al., 2015). TGFβR1 and -2 were identified as putative miR-181c targets (He et al., 2016). Overexpression of miR-181c reduced TGF-β signaling through TGFβR1 and TGFβR2, reducing cell migration (He et al., 2016). Dysregulation of miR-181c also contributes to the aggressiveness of osteosarcoma and regulation of TGF-β signaling through targeting SMAD family member 7 (SMAD7), which negatively regulates TGF-β (Fu et al., 2019). This subsequently promoted epithelial mesenchymal transition (EMT) of tumour cells, affecting cell plasticity and facilitating the initial phase of tumour cell migration.

In addition, the transcription of miR-181c itself has also been found to be regulated in a negative feedback fashion by the main TGF-β transcription factors, SMAD2/3 (Redshaw et al., 2013). Transcription of pri-miR-181c/d was shown to be dependent on SMAD2/3 activation in embryonic mouse stem cells and early embryos (Redshaw et al., 2013). This indicates a strong negative feedback relationship between miR-181c and TGF-β signaling that could have many implications in diabetes-impaired responses due to the vast and multifaceted reach of this signaling pathway. The regulation of miR-181c and its control over TGF-β intermediates and signaling molecules is yet to be investigated in the context of diabetes-impaired angiogenesis but may offer a novel treatment paradigm for vascular complications in diabetes (Figure 2).

### Mitochondrial Function and Metabolism

Studies have shown that while the HIF-1α/VEGFA signaling axis is important, cellular pathways that drive endothelial dysfunction including cellular metabolism, mitochondrial function and oxidative stress, also contribute to diabetic vascular complications (Kolluru et al., 2012). T2DM is known as a metabolic disorder, characterised by insulin sensitivity and an inability to appropriately uptake and metabolise glucose. Cellular metabolism is important in maintaining the energy requirements of the cell. The control of metabolic homeostasis requires a balance between the generation of adenosine triphosphate (ATP) via aerobic versus anaerobic respiration and the regulation of pathways that consume ATP (Zhang et al., 2009). While there are several biological pathways that control metabolic homeostasis, of particular interest in T2DM is the master metabolic regulator adenosine monophosphate-activated protein kinase (AMPK), which is activated in response to low cellular energy levels (Shrikanth and Nandini, 2020). Its activation promotes upregulation of ATP producing processes (fatty acid oxidation and glycolysis) and downregulation of ATP consuming processes (protein synthesis) (Zhang et al., 2009). AMPK is activated in response to a variety of stress stimuli, including hypoxia, oxidative stress and low energy levels. In hyperglycaemia AMPK activation is impaired and has been shown to play a causative role in the development of microvascular complications in diabetes by increasing oxidative stress and glycogen synthesis and by decreasing mitochondrial function (Shrikanth and Nandini, 2020).

Mitochondrial function is crucial in mediating aerobic respiration through oxidative phosphorylation of the main glycolysis products, pyruvate and NADH (Maechler and Wollheim, 2001). This drives the energy requirements of the cell and is critical for regulation of cellular functions, such as proliferation, migration and survival. In response to hypoxia, mitochondrial complex III generates mitochondrial ROS to stabilise HIF-1α and promote expression of angiogenic stimuli, such as VEGFA (Reichard and Asosingh, 2019). Additionally, mitochondrial DNA-deficient cells have reduced capacity to upregulate VEGFA (Satoh et al., 2011). In diabetes, mitochondrial function is impaired, and this is thought to be due to a reduction in mitochondrial biogenesis (Sivitz and Yorek, 2010).

miR-181c has also been shown to target genes involved in cellular metabolism, mitochondrial function and oxidative stress (Das et al., 2017). COX-2, a mitochondrial enzyme and downstream target of miR-181c, is induced by hypoxia and mediates VEGFA-induced angiogenesis (Wu et al., 2006). miR-181 family members are expressed in heart mitochondria (Das et al., 2012) and in cardiomyocytes miR-181c regulates the mitochondrial gene cytochrome c oxide subunit 1(mt-COX1) (Das et al., 2014). miR-181c regulation of mt-COX1 in cardiomyocytes promotes remodeling of mitochondrial complex IV, increasing ROS, while having no significant effect on mitochondrial function (Das et al., 2014). Additionally, miR-181c/d knockout reduced the infarct size following MI in mice (Das et al., 2014). Furthermore, breast cancer cell metabolism was altered by miR-181c in hypoxia (Lee et al., 2019). Induction of the glycolysis related enzymes, glucose transporter-1, hexokinase-2, pyruvate dehydrogenase kinase-1 and lactate dehydrogenase A, which are involved at various points in glucose metabolism, were impaired by miR-181c in response to hypoxia (Lee et al., 2019).

Taken together, these studies suggest that miR-181c may contribute to cardiomyocyte propensity for heart failure through regulation of mitochondrial genes and may alter the expression of enzymes important for metabolism. However, the role of miR-181c on mitochondrial function in endothelial cells and in the context of diabetes remains to be investigated.

## Axon Guidance and Angiogenesis: A Dual Role for MicroRNA-181c

Axon guidance cues are important for mediating axon and neuron maturation and promoting effective signal transfer across synapses. These guidance cues may also play a role in blood vessel navigation, facilitating the extension of blood vessels throughout coordinated networks in the body (Autiero et al., 2005; Adams and Eichmann, 2010). The spatial organisation of blood vessels throughout the body is optimised for effective delivery and removal of nutrients and waste products to and from cells (Adams and Eichmann, 2010). Nerves and blood vessels, while having extremely different functions, share anatomical similarity and are regulated, in some instances, by overlapping mechanisms (Carmeliet and Tessier-Lavigne, 2005). Precise coordination of neurovascular co-patterning and nerve formation is essential for differentiation of arteries in skin (Mukouyama et al., 2005), suggesting that these processes work together to facilitate normal nerve and blood vessel formation. Axons navigate towards target cells to form synapses through coordinated action, both locally and distantly, between attractive and repellent guidance cues and their corresponding receptors. Axons express guidance receptors at the tip of the growth cone, which is the site of elongation (Stoeckli, 2018). miR-181c is highly expressed in the brain and has been shown to target axon guidance molecules to facilitate axon and dendrite extensions (Kos et al., 2016; Chen et al., 2017). Taken together, this suggests that there may be a link between axon guidance and angiogenesis that could provide some insights into miR-181c action.

Neuropilin-1 (NRP1) is a transmembrane receptor that has a dual role in axon guidance and blood vessel navigation (Lampropoulou and Ruhrberg, 2014). NRP1 mediates axon extension within the spinal cord and brain and contributes to blood vessel navigation by interacting with VEGFA, promoting migration of endothelial cells during angiogenesis. This is mediated through dual expression of NRP1 and VEGFR2 (Lampropoulou and Ruhrberg, 2014). Retinal ganglion axons and endothelial cells require NRP1 for normal axon organisation and vascular co-patterning (Erskine et al., 2017). NRP1 interacts with semaphorins, (SEMA) -A, -B, -C, -D, -F and can act as a VEGF receptor in both endothelial cells and neurons. NRP1 can also interact with VEGF receptors, VEGFR1 and VEGFR2, as well as other angiogenic signaling molecules, such as PDGF. This increases the binding affinity of VEGF to VEGFR2 by acting as a co-receptor, resulting in increased signal transduction and endothelial cell chemotaxis (Herzog et al., 2011).

Semaphorins are repulsive axon guidance molecules that activate NRP1 as well as other guidance receptors, such as plexins (Nasarre et al., 2014). The 3’UTR of SEMA4 contains a miR-181c binding site, and recently has been confirmed as a target for miR-181c (Chen et al., 2017). The functional implications of this, however, are yet to be explored. Semaphorins are extremely important during development of the central nervous system, and their knockdown results in severe defects (Pasterkamp and Verhaagen, 2006). Semaphorins and their receptors are re-expressed upon skin injury and may play a role in promoting nerve regeneration and angiogenesis during wound healing. During wound healing newly formed vasculature also acts as a scaffold for the projecting axons, which in conjunction with guidance cues such as VEGFA and HIF-1α, that are upregulated in response to hypoxia in the wound environment, guide nerve innervation within the healing wound. This highlights some of the potential consequences of miR-181c regulation of SEMAs on the regulatory networks that exist during wound healing. However, this needs further investigation.

Studies have shown that knockdown of these guidance molecules or their receptors results in vascular defects that are characterised by changes in vascular growth, size, function, and patterning during development (Autiero et al., 2005). These findings suggest that the formation of nerves and blood vessels is a coordinated process. Functionally impaired or damaged neural circuits are thought to lie at the center of neurodegenerative diseases like Parkinson’s Disease and Alzheimer’s Disease (AD) (Stoeckli, 2018). Neurodegenerative diseases are characterised by damage to the central nervous system caused by inflammation (Friese et al., 2014) or impaired clearance of intracellular inclusion bodies (Park et al., 2020). It has become increasingly evident that impaired angiogenesis underlies the pathophysiology of many neurodegenerative diseases (Zacchigna et al., 2008). We therefore postulate that the crosstalk between angiogenic signaling, and axon guidance cues may be important for understanding the mechanisms surrounding these diseases and could provide alternative targets for therapeutic intervention or to predict disease progression.

Levels of miR-181c were shown to be dysregulated in the brains of patients with AD and other neurological conditions that are characterised by damaged neural circuits, impaired angiogenesis, and neuron death (Cogswell et al., 2008; Martinez and Peplow, 2020). Changes to circulating miR-181c levels are also seen in the serum of patients with AD (Manzano-Crespo et al., 2019), suggesting that altered miR-181c regulation in the brain may contribute to neurodegenerative diseases, or perhaps is a resulting effect of the disease itself. miR-181c is highly expressed in the cerebellar cortex and is thought to play an important role in neuron and axon development (Kos et al., 2016). Much like what we have previously shown in mouse hindlimbs, miR-181c is also transiently regulated in response to ischaemia in the brain. In response to ischaemic stroke, miR-181c levels are rapidly increased in the serum of patients (Ma et al., 2016). Additionally, miR-181c negatively regulates neuroinflammation of microglia through the toll-like receptor 4 (TLR4) in response to ischaemia (Zhang et al., 2015). This suggests that transient modulation of miR-181c is important for mediating acute survival responses to hypoxia in the brain.

Following an ischaemic event there is an immediate upregulation of genes triggered by these imbalances in oxygen supply and demand. These genes converge on signaling pathways that promote re-oxygenation of the ischaemia site through angiogenesis (Liu, 2015) but also promote new axon growth to re-form any damaged neuronal circuits in the brain (Hinman, 2014). Sequencing studies of the brain have revealed that miR-181c may target several genes that are important in these processes (Kos et al., 2016). Upon inhibition of miR-181c in neurons, neurite sprouting is increased (Kos et al., 2016). This is attributable to not only an increase in branch number but to the development of higher-order branches that are characterised by longer axon length and more intersecting dendrites, giving rise to more complex neurite networks. Additionally, overexpression of miR-181c resulted in neuron death in response to cerebral ischaemia by reducing expression of c-Fos and downstream targets AP-1 and NFATc1 (Meng et al., 2020). Indicating that miR-181c regulation may contribute to downstream hypoxia-responsive cellular process that mediate axonal guidance and cell survival in the brain.

Tripartite Motif Containing 2 (TRIM2), a ubiquitin ligase that contributes to normal brain function, is dysregulated in AD and other neurodegenerative diseases and knockdown of TRIM2 in mice results in severe neurodegeneration (Li J. J. et al., 2020). TRIM2 has been identified as a putative miR-181c target in the brain (Schonrock et al., 2012). Interestingly, in a study conducted using rats that had undergone carotid artery ligation, TRIM2 levels were upregulated in the hippocampus following cerebral hypoperfusion (Fang et al., 2017). The expression of TRIM2 was inverse to miR-181c expression which was reduced in response to hypoperfusion in the hippocampus, suggesting an inverse relationship between miR-181c and TRIM2 regulation in response to hypoxia (Fang et al., 2017). Furthermore, miR-181c overexpression increased dendrite branching and spine density leading to an improvement in cognitive impairment that was attributed to miR-181c-mediated inhibition of TRIM2. TRIM2 may also have a prominent role in angiogenesis. Knockdown of TRIM2 impaired both inflammatory-driven and ischaemia-mediated tubule formation of HCAECs (Wong et al., 2018).

Collectively, these studies suggest a complex role for miR-181c in the formation of axonal and dendrite projection suggesting divergent roles that are highly dependent on location and intracellular environment. These studies show that miR-181c targets molecules involved in angiogenesis and cell survival within the brain in response to hypoxia and that dysregulation of miR-181c may contribute to neurodegenerative diseases that are often associated with impaired angiogenesis. Highlighting a potential role for miR-181c in angiogenesis across multiple disease pathologies.

## Conclusion

We have previously identified a novel anti-angiogenic role for miR-181c and in this review have highlighted how this miRNA may be dysregulated in diabetes. miR-181c expression is altered in response to hypoxia, suggesting that its regulation may be important for mediating ischaemia-driven angiogenesis in diabetes. We have highlighted some of the pathways and molecular targets that miR-181c has been shown to regulate that are important for angiogenesis. These include either direct or indirect regulation of critical angiogenesis mediators, such as HIF-1α and VEGA, as well as molecules involved in processes such as basement membrane degradation, cellular proliferation, invasion and migration, mitochondrial function as well as tissue remodeling and maturation. These processes work in a time-dependent and cohesive manner to facilitate normal blood vessel growth in response to hypoxia.

In addition, miR-181c is dysregulated in diseases in which angiogenesis becomes dysfunctional, such as in cancer and neurodegenerative diseases such as AD. In these varying cellular contexts, miR-181c expression is also regulated by hypoxia and, in the brain, miR-181c targets molecules that have dual roles in axon guidance and blood vessel growth and navigation. Additionally, circulating levels of miR-181c may be predictive of severe diabetic macrovascular complications in different populations and could be used to inform therapeutic intervention in these patients. Taken together, this evidence highlights that miR-181c may be an important mediator of ischaemia-driven angiogenesis in diabetes and that it may be a beneficial therapeutic target for orchestrating the multiple factors required for adequate stimulation of angiogenesis in a clinical setting.
